# Exploring pathways to optimise care in malignant bowel obstruction (EPOC): Protocol for a three-phase critical realist approach to theory-led intervention development for shared decision-making

**DOI:** 10.1371/journal.pone.0294218

**Published:** 2024-01-25

**Authors:** Alison Bravington, Jason W. Boland, Sarah Greenley, Michael Lind, Fliss E.M. Murtagh, Michael Patterson, Mark Pearson, Miriam J. Johnson

**Affiliations:** 1 Wolfson Palliative Care Research Centre, Hull York Medical School, University of Hull, Hull, United Kingdom; 2 Hull York Medical School, University of Hull, Hull, United Kingdom; PLOS: Public Library of Science, UNITED KINGDOM

## Abstract

**Introduction:**

Malignant bowel obstruction is a distressing complication of cancer, causing pain, nausea and vomiting, and often has a poor prognosis. Severe and rapidly developing symptoms, a lack of robust clinical guidelines and the need for multidisciplinary input make treatment decision-making challenging. Sharing decision-making with people with malignant bowel obstruction and their caregivers can be difficult, and inconsistent communication creates serious deficiencies in care by amplifying patients’ distress and uncertainty. Little attention has been paid to the implicit influences on this process–for example, the role of discipline-related norms and the beliefs of decision-makers. This study will explore how these processes work and develop interventions to improve shared decision-making.

**Methods and analysis:**

Exploring Pathways to Optimise Care (EPOC) is a three-phase study set within a critical realist framework: (i) realist review, to develop explanatory theory describing collaborative decision-making in the management of malignant bowel obstruction; (ii) an in-depth interview study using modified grounded theory to explore the active ingredients of this collaboration in practice settings; and (iii) the presentation to stakeholders (practitioners, patients and caregivers) of integrated results from Phase I (theory developed from the literature) and Phase II (theory developed from current practice) as a basis for intervention mapping. We aim to produce recommendations to address the challenges, and to develop a set of tools to (i) aid interaction around shared decision-making and (ii) aid interprofessional interaction around the management of this condition.

**Registration details:** The realist review is registered with PROSPERO (CRD42022308251).

## Introduction

Malignant bowel obstruction–a blockage of the intestines by cancer–can present at diagnosis, or at any time during the cancer journey. It may be due to operable localised cancer, or inoperable invasive or disseminated cancer. Overall, around 15 per cent of all cancer patients are affected [1], but bowel obstruction is most common in ovarian cancer (about half of all patients) and colorectal cancer (just over a quarter) [2, 3]. Bowel obstruction has a major impact on patients and their families: symptoms include severe nausea and/or abdominal pain and vomiting (which may be persistent, large-volume and/or faeculent), and are distressing to experience and to witness [4]. While some local NHS Trusts have produced guidelines for management of the condition, these emerge from clinical experience rather than a broad evidence-base including patient perspectives. There are variations in clinical practice between units, and attention to quality of life is lacking [5].

Working out the best way to treat malignant bowel obstruction is challenging. Patient journeys are variable [6], and can involve intense periods of episodic care across trajectories of obstruction, resolution, and recurrence. Management can include surgery, stenting (sometimes as a bridge to surgery), corticosteroids, somatostatin analogues, and total parenteral nutrition. Systematic reviews report substantive ongoing uncertainties regarding management [7–11]. Curative surgery may sometimes be possible, and palliative surgery may improve survival for some [12]; in others, surgery may be ineffective, unsafe, or inconsistent with broader goals of care [13, 14]. A Cochrane Clinical Answer in 2018, drawing on Cousins et al’s 2016 review [7], stated that “no conclusions as to the benefits or harms of surgery for malignant bowel obstruction due to gynaecological or gastrointestinal cancer can be drawn” [15].

The need for a better evidence-base to support clinical decision-making is undisputed [16], but there is another serious deficiency in the clinical care of patients with malignant bowel obstruction. Recent research demonstrates that inconsistent communication between practitioners and patients can create and amplify distress and uncertainty–patients and their families often receive conflicting information and advice, sometimes during the same episode of care [17, 18]. Patients with malignant bowel obstruction and their caregivers have highlighted that information about treatment and possible pathways of care should be better tailored to their needs, and the patient voice better represented. These issues need to be sensitively and comprehensively addressed to ensure that face-to-face communication is consistent with the NIHR’s service delivery recommendations [19], and to ensure optimal treatment decision-making that maximises survival *and* quality of life.

In the past two decades, shared decision-making (SDM) between clinicians and patients has become a cornerstone of person-centred care [20, 21]. SDM is most often defined as ’the process of integrating both the best available evidence and patients’ values and preferences in the decision-making process’ [22]. SDM in practice is more complex, involving the communication of possible treatment options and the potential outcomes, probabilities and uncertainties by the clinician, and the processing of this information by the patient–followed by the weighing of the benefits and harms of treatment against the patient’s values and social context [23]. However, multidisciplinary, collaborative care is more complex than the dyadic communication typically characterised by SDM, making it necessary to consider roles and relationships (between practitioners, and between practitioners, patients and their families [24]) and how they influence decision-making.

Malignant bowel obstruction is an example of a complex health condition. Its mode of presentation, pace of progression, and the range of disciplinary collaboration required can make management of the condition challenging, and decision-making in true partnership with patients and their families may not be possible at every point in its trajectory [23]. Where decisions about emergency symptom control, the benefits of surgery, the likelihood of recurrence and/or end of life care combine, SDM becomes highly complex. Given that patient perspectives on decision-making in palliative care in particular are under-researched [22, 25], there is currently little evidence to help conceptualise how decision-making partnerships between multidisciplinary teams and people with malignant bowel obstruction work, from the first occurrence of an obstruction to end of life care.

Attempts to conceptualise SDM suggest a range of underlying social influences, such as clinicians’ perceptions of patients’ capacity [26], patient and practitioner values and motivations [27], and patients’ perceptions of their own validity as decision-makers [25]. These implicit beliefs and behaviours reflect the ‘active ingredients’ of health care practice that are critical to the success of complex interventions [28]. In the language of realist synthesis [29, 30], they are ‘mechanisms’ [31], affected by a range of contextual issues such as practitioner-patient relationships, quality of information provision, continuity of care, and cultural sensitivity [23]. How these mechanisms work in relation to complex health conditions can only be explored by moving from the general (theoretical conceptions of how mechanisms produce outcomes in a social process such as decision-making) to the particular (how these theoretical causal pathways operate in specific contexts).

### Aims

We aim to: i) improve understanding of communication around treatment decision-making in the management of malignant bowel obstruction, ii) produce recommendations to address the challenges, and iii) with the input of patient, caregiver and practitioner stakeholders, develop a set of decision tools to aid interaction around shared decision-making and interprofessional interaction around the management of this condition.

## Methods and analysis

### Epistemological approach

EPOC will use an innovative methodological approach to explore the process of decision-making and inform intervention development, underpinned by a critical realist paradigm. Critical realism draws on both positivist and constructivist paradigms to explain social events and their potential relationship with ‘real world’ policy and implementation [32, 33]. It sits in the gap between qualitative, social constructionist descriptions of multiple perspectives on problematic issues and the scientific analysis of cause-and-effect based on what we can observe [34, 35]. Critical realism acknowledges a reality independent of social constructions, but recognises and works with both observable and unobservable elements of this reality. This allows the theorisation of chains of cause and effect that include invisible processes (such as trust, or perceptions of capacity), and the exploration of how implicit structural and social processes shape human action and flex and reconfigure as their surrounding context changes.

### Study design

This three-phase study is designed to explore implicit and explicit mechanisms and outcomes of collaborative decision-making involving multiple clinical disciplines, patients and caregivers, as a basis for stakeholder-led intervention mapping [36]. It will address the following research questions:

- How, when and in what circumstances can decision-making be shared in the management of malignant bowel obstruction?
- How might patients’, caregivers’ and clinicians’ experiences of treatment decision-making differ across a range of contexts?
- What constrains and enables the sharing of decision-making in practice?
- What are the core components of consistent shared and/or patient-centred decision-making in malignant bowel obstruction, and how can we operationalise these to improve decision-making communication?

#### Phase I: Realist review

A literature review using realist synthesis will be used to develop and test a theory of shared decision-making in the management of malignant bowel obstruction (PROSPERO registration: CRD42022308251) through iterative searches of specialist databases, grey literature and web resources undertaken by the research team with the assistance of an information specialist (SG). The focus of the searches will be informed by stakeholder consultation during the course of the review–stakeholders include practitioners involved in the management of malignant bowel obstruction (surgeons, oncologists, dietitians, palliative care consultants, specialist nurses), patients and their caregivers. Studies will be assessed on their rigour and relevance to theory development and the review will be reported following RAMESES guidance [37]. Synthesis will involve inference of a series of hypothetical context-mechanism-outcome chains related to the sharing of decision-making in the management of malignant bowel obstruction to explore what factors might facilitate or constrain this process, expressed in the form of a programme theory (an explanatory model of the shared decision-making process). The programme theory will be iteratively revised during the course of the review [37, 38].

#### Phase II: Interview study

An interview study will be used to develop a theory of shared decision-making in malignant bowel obstruction as it happens in current practice. In-depth interviews (n∼80) will be conducted with patients, caregivers and practitioners recruited across five secondary care centres and four specialist palliative care units in the north of England. Governance applications are being conducted parallel to Phase I; initial recruitment began at Site 1 in March 2023 and is due to begin across all sites in October 2023; Phase II will complete at the end of August 2024.

The sample size is provisional, estimating data saturation based on the theory-building, inductive approach to analysis described below [39], the range of stakeholders included and the number of study sites. Local clinical collaborators will contact practitioners, patients and caregivers with experience of managing malignant bowel obstruction with study information sheets. Patients and caregivers will be approached by the clinical team where the patient is judged stable enough to participate without unacceptable burden; bereaved caregivers will be approached at least eight weeks after the patient’s death. Written informed consent will be obtained by the researcher before commencing each interview.

The interview guide begins with introductory questions to establish rapport and explore experiences of decision-making in malignant bowel obstruction. The participant-led graphic elicitation technique, Pictor [40, 41], is then introduced to elicit a narrative of a particular episode or series of episodes of communication around decision-making in malignant bowel obstruction, focused on an example of the interviewee’s choice. Pictor uses arrows to represent roles, relationships and interactions in the form of a diagram or ‘chart’, and has a history of use in encouraging rich data in research exploring collaborative processes in health care, including palliative care [42–44]. The technique has been chosen for its utility in representing complex events and/or episodic care in simple terms, and its focus on capturing the ‘invisible’ social mechanisms which influence communication [41]. Practitioners will be asked to focus on experiences of a specific patient, and patient and caregiver interviewees will be asked to focus on personal experiences. The technique does not require the use of names or other identifying details. If an interviewee does not wish to create a Pictor chart, they will be offered an in-depth qualitative interview without diagramming. Charts will be anonymised where necessary, and photographed; a simple content analysis framework will be used to explore the frequency of appearance of stakeholder roles, and to compare chart structure [41].

All interviews will be audio-recorded, transcribed and anonymised. Analysis will be conducted using modified grounded theory. Grounded theory techniques have been identified in recent work for their potential alignment with critical realist approaches, but remain diverse in their application [45–49]. The active ingredients of decision-making communications in the management of malignant bowel obstruction will be mapped in four steps (adapted from Hoddy, 2019 [48]), through: (i) the identification of its core components, (ii) the identification of demi-regularities or patterns in the process, (iii) abduction (theorising patterns of cause and effect that bring about these regularities), and (iv) retroduction (examining how these patterns might be affected by fluctuations in relational or organisational contexts). An explanatory model of shared decision-making in malignant bowel obstruction will be developed for comparison with the theory developed from evidence in Phase I. Decision maps will be drafted as part of the analytic process: key decision points and associated theories of cause and effect will be identified across a range of treatment trajectories to address communication challenges and identify objectives for change.

#### Phase III: Stakeholder-led intervention development

The aim of Phase III, which will begin in September 2024, is to develop a set of decision tools for malignant bowel obstruction: (i) to aid interaction around shared decision-making between patients, caregivers and practitioners, and (ii) to aid interprofessional interaction around the management of malignant bowel obstruction.

*Patient and Public Advisory Group meeting*: Results of Phases I and II will be presented in plain language to a patient and public advisory group convened for the study to invite feedback. This consultation will also contribute to the development of clear written and visual material for presentation at subsequent stakeholder workshops.

*Stakeholder intervention development workshops*: Two sequential intervention development workshops will be conducted face-to-face or online, depending on stakeholder preference: i) with practitioners and ii) with patients and caregivers. We will retain patient, caregiver and practitioner stakeholders from Phases I and II where possible, and publicise the workshops through UK cancer charity partners to identify additional members. Decision maps produced from Phases I and II will be presented to trigger reflection and dialogue around the suitability of their components [50] and their overarching comprehensibility and usability [51].

### Data management and archiving

Anonymised audio, text and visual methods data will be managed in accordance with University of Hull Data Management guidelines and will be available only to members of the research team or to the relevant university authority for audit and governance purposes. Personal contact data for stakeholders and interviewees will be kept in a password protected Excel file accessible only to the Research Team and Study Administrator. Written consent forms will be kept in a locked cabinet at Hull York Medical School (Allam Medical Building, University of Hull).

### Project management and public involvement

The study will be overseen by a monthly Project Management Group and six-monthly external Study Steering Committee, and will be subject to audit/monitoring according to study sponsor, site R&D and funder requirements. Both oversight groups will include clinicians with experience of bowel obstruction and research methodologists. The project will adhere to UK Standards for Patient and Public Involvement [52]. The Study Steering Committee will include a Patient and Public Involvement (PPI) representative with experience of malignant bowel obstruction, and a bi-annual, study-specific Patient and Public Advisory Group will be convened to advise the Project Management Group. Patient-facing documentation will be shared for comment with study PPI representatives and an established independent faculty-level PPI group prior to its release.

### Ethical approvals and study registration

Ethical approvals were obtained on 11^th^ July 2022 from North West–Greater Manchester East Research Ethics Committee, reference 22/NW/0153, and approval was received from the Health Research Authority on 12^th^ July 2022. Informed consent to participate will be taken in writing for all Phase II interviews. The Phase I review is registered with PROSPERO (CRD42022308251).

### Study status and timeline

**Fig 1:**
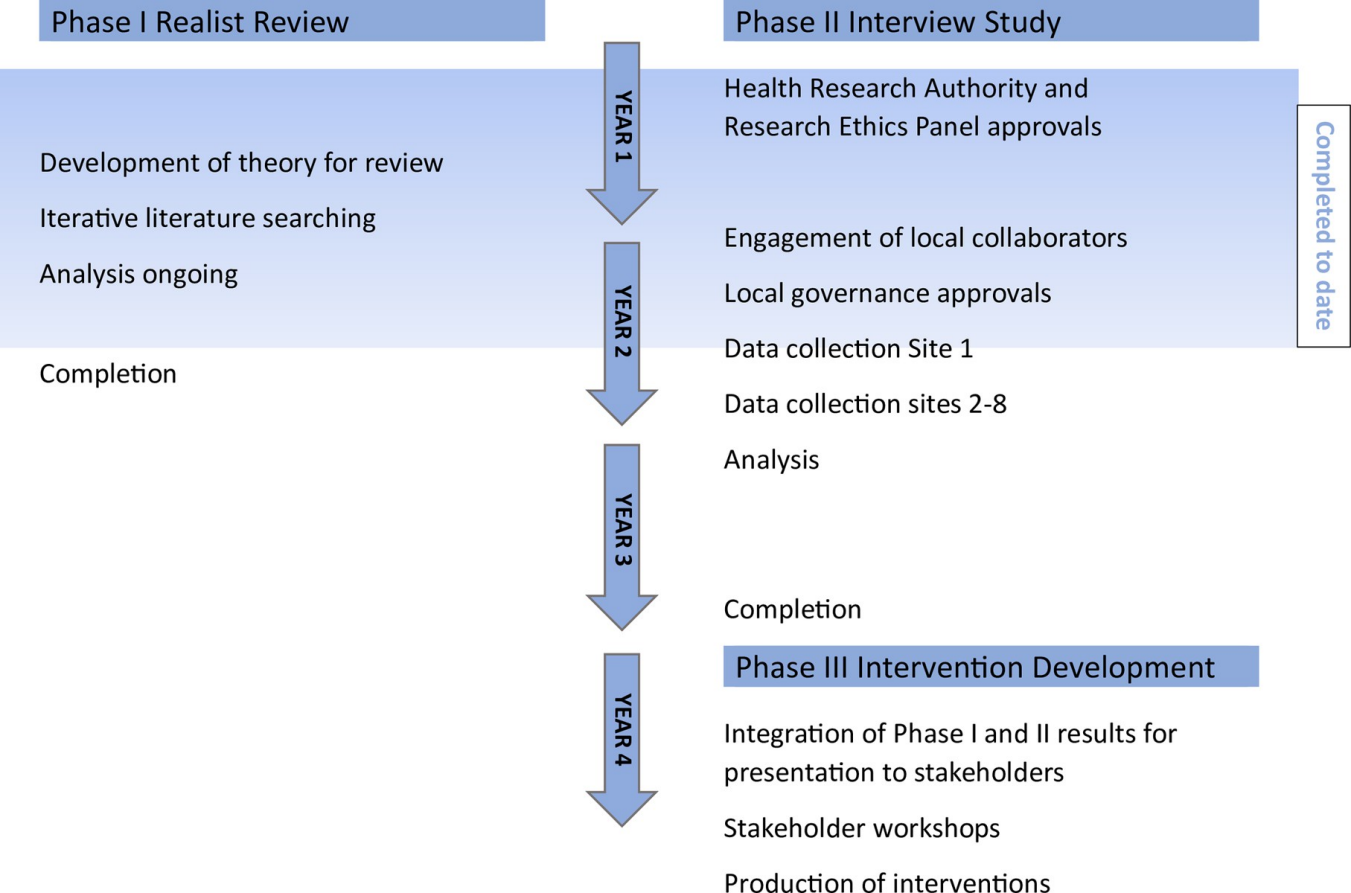
Study status and timeline.

Analysis of the review commenced alongside governance applications for the interview study, but is not yet complete. The study timeline is illustrated in Fig 1.

## Discussion

### Strengths and limitations of the study

The EPOC Study uses a unique combination of methods, underpinned by a critical realist approach, to facilitate the integration of evidence, theory and practice in a way which will inform improvements in the management of a complex health condition. Stakeholders including clinicians, patients and caregivers will be involved in all phases of the study, which adheres to UK Patient and Public Involvement standards. Intervention development will be guided by stakeholder consultation. The study focuses on communication and decision-making in the management of a malignant bowel obstruction, but may demonstrate transferability to other areas of applied health research exploring complex conditions. The research is taking place in a UK setting in the North of England, but its focus on the contextual details of service delivery may provide guidance applicable to other settings.

### Dissemination

Dissemination of overarching findings and intervention frameworks will be conducted via research centre networks, through clinical networks at study sites, and through connected professional networks and social media. Stakeholder organisations (such as dietitian or oncology associations) will be contacted with a summary of the study results and electronic links to patient and practitioner interventions. Throughout the study, interim findings will be disseminated to involved stakeholders, applications for posters and oral presentations will be made to national and international academic conferences, and academic papers will be submitted to high impact journals. Lay summaries will be produced for dissemination to relevant PPI groups and UK cancer charities, and published on the Wolfson Palliative Care Research Centre website and the Yorkshire Cancer Research website.

## Supporting information

S1 File EPOC original study protocol submitted to ethics panel and health research authority. (PDF)

S2 File EPOC Draft interview guides. (PDF)
