# Supplementary material for: Exploring pathways to optimise care in malignant bowel obstruction (EPOC): Protocol for a three-phase critical realist approach to theory-led intervention development for shared decision-making
Source: PLoS One. 2024 Jan 25;19(1):e0294218. doi: 10.1371/journal.pone.0294218 (PMC10810450; doi:10.1371/journal.pone.0294218)
Supplement: S1 File — (PDF) [file pone.0294218.s001.pdf]

DRAFT INTERVIEW GUIDE

PARTICIPANTS WITH BOWEL OBSTRUCTION

Complete informed consent process prior to interview.

Introductory information

1. Thank you for taking part in this study, it's very valuable to us to hear you talk about your experiences. *[Explain briefly what the study is about, that it is focusing on communication and decision-making around treatment for a bowel blocked by cancer. Offer the opportunity to ask any further questions.]*

2. The interview usually takes about an hour, and what we'll do is begin with a general chat about your diagnosis and the things that have been happening to you recently. Then we're going to do a simple visual exercise using some arrows and a piece of paper, which will help you to think about the care you've received from health services and other people. This is a way of mapping out what's happened to you without you having to think too hard, and it will help me to ask questions about what happened to you, rather than general questions. Before we do this, I have a couple of introductory questions:

a. Please could you tell me a little bit about what happened when you first had symptoms of a blockage in your bowels, and how you got help?

b. What kind of decisions did you have to make about things during your treatment/care? [If none, did they need to make any decisions at all during this episode of care?]

3. PICTOR: What we're going to do next is try the visual exercise – I'll guide you through this step-by-step, if there's anything that doesn't make sense to you please do say.
[Explain Pictor]
First of all, think about all of the people and/or organisations that were involved in your care, particularly the people who helped you through the decisions about treatment – so this is not just doctors and nurses, you can include anyone who you feel was significant to you during this time. Write these people on to the arrows, a separate arrow for each. It would be great if you could write people's role on the arrow rather than their name (e.g. surgeon, GP, husband/wife, friend etc). If you want to use an arrow to represent a group or a place or something else, please do whatever makes sense to you. Please make sure you write an arrow to represent yourself.
[Wait until arrows done, all though more can be written and added at any stage.]

Now I'd like you to arrange the arrows on this big piece of paper in a way that tells the story of that moment in your care. People usually use the direction of the arrows, or the distance between them, to say something about how people supported or helped them, or perhaps weren't so helpful or supportive. There's no right or wrong way to do this – just lay the arrows out in a way that makes sense to you. Do you have any questions?
[Show an example of a Pictor chart, if necessary.]

I'm going to leave you to do this on your own for ten minutes, unless you'd rather I stayed?
[5-10 minute break for participant to complete chart.]

4. Thank you for doing this. It would be great if you could explain why you've arranged the arrows in this way – tell me the story behind this.

[When the story of the Pictor chart has been told, further probe questions will steer towards exploring decision-making points in the events represented in the Pictor chart, and aspects of collaboration – for example, questioning why arrows are close together or far apart or pointing in the same or different directions, which roles seem to be missing and why, and asking about the general shape of the chart (for example, it may be circular to represent the patient at the centre, or arranged in lines to represent the passage of time). Questions might include: Who made the final decisions about what to do? How did you feel about this process as a whole? Who, out of the people shown here, was most important in helping you get your thoughts straight at this moment? Is there anyone here who could have given you better help or support? What information did you need? Who supplied this?]

5. Is there anyone or anything missing from this chart – people who you felt should be involved in helping you at this time, but weren't? This can be for any reason at all. Can you write them onto arrows too? Can you add them to the chart to show me how or where you would have liked them to be involved? [This may be combined with Q7 after piloting.]

6. If you could have changed the way things happened, what would have happened differently? *[Participants sometimes want to change the position of the arrows to represent a better scenario, and will be encouraged to do so if they wish, explaining what they are changing and why.]*

7. How are you now? Have your healthcare team told you what happens next?

8. Is there anything I should have asked you, but I haven't?

Thank you and close interview, ensure contact details for research team are available.

DRAFT INTERVIEW GUIDE

CAREGIVERS

Complete informed consent process prior to interview.

Introductory information

1. Thank you for taking part in this study, it's very valuable to us to hear you talk about your experiences. *[Explain briefly what the study is about, that it is focusing on communication and decision-making around treatment for a bowel blocked by cancer. Offer the opportunity to ask any further questions.]*

2. The interview usually takes about an hour, and what we'll do is begin with a brief chat to understand a little of the background around your experiences of looking after someone with a blocked bowel. Then we're going to do a simple visual exercise using some arrows and a piece of paper, which will help you to think about the care *[your significant person/name of patient]* received from health services and other people and what this felt like to you, as a caregiver. This is a way of mapping out what's happened without you having to think too hard, and it will help me to ask questions about your situation, rather than general questions. Before we do this, I have a couple of introductory questions:

   a. Please could you tell me a little bit about what happened when *[name]* first had symptoms of a blockage in their bowels? How did the situation feel for you?

   b. What decisions were made about what kind of treatment to have?
   Did you take part in the decision-making?

3. PICTOR: What we're going to do next is try the visual exercise – I'll guide you through this step-by-step, if there's anything that doesn't make sense to you please do say.
[Explain Pictor, adjusting the technique to suit the participant where helpful]
First of all, think about all of the people and/or organisations that have been involved in some way in helping you manage *[patient's]* bowel blockage and make decisions about their treatment and care – not just doctors and nurses, you can include anyone who's been significant to you during this time. Write these people on to the arrows, a separate arrow for each. Try to write people's role on the arrow rather than their name (e.g. surgeon, GP, pharmacist, friend etc). If you want to use an arrow to represent a group or a place or something else, please do whatever makes sense to you. Please make sure you write an arrow to represent <u>yourself</u>, and an arrow to represent *[name of patient]*.
[Wait until arrows done, all though more can be written and added at any stage.]

Now I'd like you to arrange the arrows on this big piece of paper in a way that tells the story of what happened. People usually use the direction of the arrows, or the distance between them, to say something about how people were supportive, or perhaps weren't so supportive. There's no right or wrong way to do this – just lay the arrows out in a way that makes sense to you. Do you have any questions?
[Show an example of a Pictor chart, if necessary.]

I'm going to leave you to do this on your own for ten minutes, unless you'd rather I stayed?
[5-10 minute break for participant to complete chart.]

4. Thank you for doing this. It would be great if you could explain why you've arranged the arrows in this way – tell me the story behind this.

[When the story of the Pictor chart has been told, further probe questions will steer away from tangents to investigate decision-making points in the events represented in the Pictor chart, and aspects of social interaction – for example, questioning why arrows are close together or far apart or pointing in the same/different directions, asking about specific roles, asking which roles seem to be missing and why, and asking about the shape of the chart (for example, it may be circular to represent the patient at the centre, or arranged in lines to represent the passage of time). Other questions might include: Who was helpful to you, as a caregiver, and who wasn't? Who was helpful to [patient], and who wasn't? Can you explain why? Who made the decisions about treatment? How did you feel about this process? What information did you need, as a caregiver? Who helped you out with the right information? Did [patient] understand what was happening?]

5. [Dependent on situation/bereavement]
How are things now? Have *[patient's]* healthcare team told you what happens next, in terms of further treatment or support?
OR How were things after treatment – what happened next?

6. If you could have changed the way things happened, what would have happened differently? *[Participants sometimes want to change the position of the arrows to represent a better scenario, and will be encouraged to do so if they wish, explaining what they are changing and why.]*

7. Is there anything I should have asked you, but I haven't?

Thank you and close interview, ensure contact details for research team are available.

DRAFT INTERVIEW GUIDE

CLINICIANS

Complete informed consent process prior to interview.

Introductory information

1. Thank you for taking time to help us with this study, it's very valuable to us to hear you talk about your experiences. *[Explain briefly what the study is about, that it is focusing on communication and decision-making around treatment for bowel obstruction. Offer the opportunity to ask any further questions.]*

2. The interview takes up to an hour, and what we'll do is begin with a brief chat to understand a little about your background in managing bowel obstruction patients. Then we're going to do a simple visual exercise using some arrows and a piece of paper, which will help you to think about how treatment and health care works for bowel obstruction patients. This is a way of mapping out what happens with a patient without you having to think too hard.

a. So could we begin with you telling me about your job role (your clinical background, how long you've been in this role…) ?

b. What kind of contact do you usually have with bowel obstruction patients?

3. PICTOR: What we're going to do next is try the visual exercise, which is a shorthand way of looking at a particular patient case. Usually, clinicians find this an easier and more focused way to have an interview conversation, rather than a researcher asking a list of generalised questions, and it helps to have some visuals when we disseminate the research. I'll guide you through this step-by-step, if there's anything that doesn't make sense to you please say.
[Explain Pictor]
Can you think of a relatively recent case in which you've had a complex decision to make about treatment? [By complex, I mean a decision that was shared and was complex to manage because of that, or that wasn't shared, and complex to manage because of that.]
Can you bring that situation to mind as best you can?
Now please think about all of the people who were involved, or on the periphery of that decision - people who contributed expertise or logistical support or psychosocial support, even people who were absent from decision-making who you felt should have been involved. You can include anyone at all, not just clinical staff. If you want to use an arrow to represent – a particular service or place of care rather than a person, please do – whatever makes sense to you. Please make sure you include an arrow to represent <u>yourself</u> and arrows to represent the patient and their main caregiver or caregivers.

[Wait until arrows done, all though more can be written and added at any stage.]
Now I'd like you to arrange the arrows on this piece of flipchart paper in a way that tells the story of this patient's care. People usually use the direction of the arrows, or the distance between them, to say something about how people worked together or communicated effectively, or perhaps not so effectively. There's no right or wrong way to do this – just lay the arrows out in a way that makes sense to <u>you</u>. If you would rather represent the timeline of their care with the arrows, please do. Do you have any questions?
[Show an example of a Pictor chart, if necessary. Some clinicians will choose to do something very different – e.g. fold arrows to represent nodes, or use a network or timeline layout with processes rather than people written onto the arrows. This is not questioned – the important thing is that they conceive it in a way which allows us to talk around the decision-making.]
I'm going to leave you to do this on your own for ten minutes, unless you'd rather I stayed?
[5-10 minute break for participant to complete chart.]

4. Thank you for doing this. It would be great if you could explain why you've arranged the arrows in this way – tell me the story behind this.

[When the story of the Pictor chart has been told, further probe questions will steer away from tangents to investigate decision-making points in the events represented in the Pictor chart, and aspects of social interaction/collaborative working – for example, questioning why arrows are close together or far apart or pointing in the same/different directions – and exploring the general shape of the chart – for example, why some arrows surround the patient and some are placed on the periphery, or whether a more linear layout represents a chronological process. Further probe questions might include: Who made the decisions about treatment? Which decisions were you involved in and why? What information and understandings was decision-making based on? How easy/difficult were the decisions?]

5. Did you involve the patient and caregiver in decision-making? If not, why?/If yes, how?

6. If you could have changed the way things happened, what would have happened differently? *[Participants sometimes want to change the position of the arrows to represent a better scenario, and will be encouraged to do so and explain the changes.]*

7. What happened next, with this patient? Was this what you expected?

8. Is there anything I should have asked you, but I haven't?

Thank you and close interview, ensure contact details for research team are available.
