# Supplementary material for: Exploring pathways to optimise care in malignant bowel obstruction (EPOC): Protocol for a three-phase critical realist approach to theory-led intervention development for shared decision-making
Source: PLoS One. 2024 Jan 25;19(1):e0294218. doi: 10.1371/journal.pone.0294218 (PMC10810450; doi:10.1371/journal.pone.0294218)
Supplement: S2 File — (PDF) [file pone.0294218.s002.pdf]

The EPOC Study

Exploring Pathways to Optimise Care in Malignant Bowel Obstruction

Full title: *Improving decision-making in malignant bowel obstruction: An exploration of context-specific treatment pathways and experiences to inform intervention development for person-centred care*

Version: 0.1

Date: 6th January 2022

Sponsor: University of Hull

Sponsor reference: To be confirmed.

Funder: Yorkshire Cancer Research TRANSFORM Endowment

Funder reference: HEND405AB

Start date: 1st September 2021

End date: 31st August 2024 (subsequent no-cost extension to January 31st 2025)

IRAS project ID: 308809

RESEARCH TEAM

Principal Investigators
Dr Alison Bravington
Research Fellow in Palliative Care
Wolfson Palliative Care Research Centre
Hull York Medical School,
University of Hull,
Allam Medical Building,
University of Hull, Cottingham Road
Hull, HU6 7RX

Professor Miriam Johnson
Professor of Palliative Care
Wolfson Palliative Care Research Centre
Hull York Medical School,
University of Hull,
Allam Medical Building,
University of Hull, Cottingham Road
Hull, HU6 7RX

Co-Investigators
Dr Jason Boland
Senior Clinical Lecturer/Honorary Consultant
Wolfson Palliative Care Research Centre
Hull York Medical School,
University of Hull,
Allam Medical Building,
University of Hull, Cottingham Road
Hull, HU6 7RX

Professor Michael Lind
Foundation Professor of Oncology
Hull York Medical School
University of Hull,
Allam Medical Building,
University of Hull, Cottingham Road
Hull, HU6 7RX

Dr Mark Pearson
Reader in Implementation Science
Wolfson Palliative Care Research Centre
Hull York Medical School,
University of Hull,
Allam Medical Building,
University of Hull, Cottingham Road
Hull, HU6 7RX

Professor Fliss E Murtagh
Professor of Palliative Care
Wolfson Palliative Care Research Centre
Hull York Medical School,
University of Hull,
Allam Medical Building,
University of Hull, Cottingham Road
Hull, HU6 7RX

Michael Patterson
Clinical Research Fellow
Wolfson Palliative Care Research Centre
Hull York Medical School,
University of Hull,
Allam Medical Building,
University of Hull, Cottingham Road
Hull, HU6 7RX

Information specialist for realist review:
Sarah Greenley
Research Fellow
Hull York Medical School
University of Hull,
Allam Medical Building,
University of Hull, Cottingham Road
Hull, HU6 7RX

SPONSOR

The University of Hull will act as sponsor for the study. Delegated responsibilities will be assigned to the Universities and NHS trusts taking part in this study.

The University of Hull will be responsible for, and administer the financial aspects of, the grant. The University of Hull has arranged insurance to cover the legal liability of the University as Research Sponsor in the eventuality of harm to a research participant arising from management of the research. For participants recruited at NHS sites, NHS indemnity scheme/NHS professional indemnity will apply with respect to claims arising from harm to participants at site management organisations.

FUNDING

This study is funded by Yorkshire Cancer Research, grant reference number: HEND405AB.

COMPLIANCE

This study will be conducted in compliance with the protocol, the Declaration of Helsinki (South Africa, 1996), the Research Governance Framework for Health and Social Care, Data Protection Act 2018 and General Data Protection Regulations (GDPR) 2018, and other regulatory requirements as appropriate.

ABBREVIATIONS AND GLOSSARY

HCP	Health care professional
IMBO	Inoperable malignant bowel obstruction
MBO	Malignant bowel obstruction
SDM	Shared decision-making
WPCRC	Wolfson Palliative Care Research Centre
YCR	Yorkshire Cancer Research

CONTENTS

1. Project Summaries....6

2. Background....7

3. Aims and Objectives....10

4. Study Design....11

Phase 1: Realist review....11

Phase 2: Patient trajectories interview study....15

Phase 3: Intervention development....19

5. Project Management....20

6. Patient and Public Involvement....20

7. Ethical and Regulatory Approvals....20

8. Data Management and Archiving....22

9. Study Timeline....23

10. References....24

1 PROJECT SUMMARIES

Lay Summary: About one in six people with cancer experience the blockage of their intestines by a tumour (malignant bowel obstruction). This stops people eating and drinking, causing severe pain, nausea and vomiting. Making decisions about treatment can be difficult – there are no nationally agreed guidelines. Involving patients in decision-making can also be difficult where their symptoms are severe, or their cancer is very advanced. The aim of this project is to inform decision-making about treatment pathways, examine how these decisions can be shared with patients, and facilitate the right choice of treatment. We will review existing evidence to guide an interview study with patients, caregivers and clinicians exploring how treatment decisions are made. The last stage of the project will involve discussing the results of our review and interview study with patients, caregivers and clinicians to co-design patient and caregiver information and on-line training for health care professionals.

Technical Summary: Fifteen per cent of people with cancer experience malignant bowel obstruction (MBO). This prevents eating and drinking and causes distressing symptoms, including pain, intense nausea and distressing vomiting, needing urgent management. Various management options are available, from surgery or stenting to less invasive methods such as medication or tube-drainage of intestinal contents. Every patient's situation is unique, making clinical decision-making difficult. Surgery gives the best option for longer-term survival in the some patients, but there are currently no nationally agreed guidelines and practice varies widely nationally. High-level evidence to support clinical decision-making is lacking, and our research shows that patients feel burdened with and distressed by uncertainties aggravated by conflicting clinical advice about best treatment. In this study we will conduct a detailed realist review of existing regional management guidelines and evidence around decision-making in MBO, exploring what works, for whom, in what circumstances. The review will be guided by input from patient, caregiver and clinician stakeholder groups. The findings will inform an in-depth interview study of patient, caregiver and clinician experiences of MBO focusing on decision-making and how this is shared. The interview study findings will inform further stakeholder workshops with

patients, caregivers and clinicians to develop patient information and on-line learning material for clinical training. Both outputs could incorporate subsequent development in the treatment evidence-base, remaining relevant.

2 BACKGROUND

Malignant bowel obstruction (MBO) – a blockage of the intestines by cancer – can present at the point of a cancer diagnosis, or at any time during the cancer journey. It may be due to operable localised cancer, or inoperable disseminated cancer. Overall, around 15 per cent of all cancer patients are affected[1], but MBO is most common in ovarian cancer (about half of all patients) and colorectal cancer (just over a quarter)[2,3].

MBO has a major impact on patients. Symptoms are distressing to experience and to witness[4,5], including severe nausea and/or abdominal pain and vomiting (which may be persistent, large-volume and/or faeculent). Where obstruction is partial, incontinence with overflow diarrhoea may occur. For clinicians, cases are memorable because of the urgent management required, witnessing patients in severe distress, and the sensory unpleasantness of the symptoms. While some local NHS Trusts have produced guidelines for the management of MBO, these emerge from clinical experience rather than a broad evidence-base including patient perspectives. There are variations in clinical practice between units, and attention to psychosocial issues and quality of life are lacking.

Working out the best way to treat MBO in individuals is challenging. Patient journeys are variable[6], involving intense periods of care across trajectories of obstruction, resolution and recurrence. Cochrane systematic reviews on treatment options (surgery[7], corticosteroids[8], total parenteral nutrition[9], somatostatin analogues[10]) report significant ongoing uncertainties regarding management. Curative surgery may be possible, and palliative surgery may improve survival for some[11]; in others surgery may be ineffective, unsafe, or inconsistent with broader goals of care[12,13]. A Cochrane Clinical Answer in 2018 stated that “no conclusions as to the benefits or harms of surgery for malignant bowel obstruction due to gynecological or gastrointestinal cancer can be drawn”[14].

In inoperable MBO, care is highly patient-specific, and there is often a mismatch of expectations between health care practitioners, patients and caregivers: goals of care can differ between patients and professionals[15,16], and the needs of patients and caregivers are not always aligned as treatment unfolds[17,18]. Our recent review of outcomes in inoperable MBO (PROSPERO registration: CRD42019150648) conducted at the Wolfson Palliative Care Research Centre (WPCRC) demonstrated that current research focuses on the technical success of interventions (for example, the resolution of obstruction, which is often not possible) and short term symptom relief, and struggles to incorporate broader issues related to quality of life.

The review also demonstrated how the choice of non-surgical treatments has broadened over the last 20 years. For some patients a stent can be used to open up the intestine, for others more conservative management (tube drainage/ symptom control/supported feeding) may be the best option in terms of quality of life[20,21]. For patients with partial obstruction, self management using dietary regimes is currently being explored but is not yet formally evidenced[19]. Where a patient cannot eat, nutrients are sometimes given directly into a vein to prevent starvation (total parenteral nutrition, or TPN). Decision-making around these treatments is not always clear-cut.

Whilst the need for a better evidence-base on which to base clinical decision-making is undisputed[22], there is another serious deficiency in the clinical care of MBO. Our current research in Yorkshire with patients, caregivers and clinicians with experience of MBO[4] demonstrates how difficulties in clinical decision-making can create and amplify distress and uncertainty. Effective multidisciplinary collaboration and good communication with MBO patients and caregivers is vital, but patients and caregivers often receive conflicting information and advice, even during the same episode of care. Our patient and caregiver participants highlighted that pathways of care and information giving should be better tailored to their needs, and the patient voice better represented, to ensure patient-centred decision-making about optimal treatment to maximise survival and quality of life – consistent with the NIHR's partnership working recommendations[23].

In the past two decades, shared decision-making (SDM) between clinicians and patients has become a cornerstone of person-centred care[24,25]. What SDM looks like in practice varies

across contexts, and where decisions about emergency symptom control, the benefits of surgery, the likelihood of recurrence and/or end of life care combine, as they do in MBO, the discussion of ways forward between clinicians and patients becomes a complex and potentially difficult process. Given that patient perspectives on decision-making in palliative care are under-researched[26], there is currently little evidence to help conceptualise how decision-making partnerships with people with MBO can evolve, from the first occurrence of an obstruction to end of life care.

Our previous research demonstrates that decision-making in partnership with patients may not always be possible where MBO presents as an emergency, and a distinction may need to be made between shared decisions and person-centred decisions. The current literature demonstrates that person-centred care may increase communication between clinicians and patients in positive ways, but this does not always lead to an improvement in the patient's quality of life or symptom burden, or lead to reduced hospital admissions[27]. Exploration of how the decision-making process around treatment relates to patient-relevant outcomes in MBO is needed in order to examine the links between the two.

Shared decision-making is most often defined as 'the process of integrating both the best available evidence and patients' values and preferences in the decision-making process'[26]. The EPOC study, Exploring Pathways to Optimise Care in MBO, will use realist methodology to develop and test a theory of shared decision-making in the management of malignant bowel obstruction. Realist philosophy provides a strategy for synthesising a broad range of evidence and providing explanations of how interventions may or may not work, taking social processes and changing contexts into account[28,29]. It advocates an ontology aligned to positivism, taking the position that direct observation in the 'real' world is an appropriate method of exploring phenomena, and an epistemology of constructionism, which acknowledges multiple perspectives on (and interpretations of) the real world. The project involves three phases. Phase 1 is a realist review, which will gather existing theoretical models of shared decision-making (SDM)[30] alongside evidence of the sharing of decision-making in the MBO management literature, policy guidelines, and other relevant documents. The aim of the review is to synthesise the evidence to produce a programme theory of SDM in MBO. In Phase 2, the programme theory will be evaluated against the

practice of decision-making in MBO through in-depth interviews with patients, caregivers and clinicians. In Phase 3, findings of the review and interview study will provide the basis of experience-based co-design[31] workshops with stakeholders (patients, caregivers and clinicians). The workshops will refine the theory of shared decision-making and use it to develop patient-relevant decision-making tools and clinical training.

The EPOC Study will gain a better understanding of the potential for shared decision-making across the multiple trajectories of MBO. The theory will explain the mechanisms or social processes which contribute to (or inhibit) patient-centred, collaborative decision-making. Feeding this understanding into stakeholder-driven intervention development will fill an urgent gap in care. It will explain how we might tailor the goals of treatment in MBO to suit the individual patient and their circumstances, how to put quality of life at the forefront of care, and how to engage patients and caregivers in this process in a way which reduces the uncertainties they experience around treatment for malignant bowel obstruction.

3 AIMS AND OBJECTIVES

The aim of this project is to improve understanding of how shared decision-making (SDM) can be achieved for patients with MBO by addressing the following objectives:

1. Map what is known about the processes and outcomes of decision-making pathways in the management of MBO.
2. Explore how decision-making about MBO treatment happens in practice, and what role patients and caregivers play in the evolution of treatment decisions.
3. Use experience-based co-design with key stakeholders to develop interventions to support shared decision-making in MBO management.

4 STUDY DESIGN

4.1 PHASE 1: Realist review of decision-making in malignant bowel obstruction

Realist reviews comprise of a set of 'systematic, theory-driven interpretative techniques, which were developed to help make sense of heterogeneous evidence about complex interventions applied in diverse contexts in a way that informs policy'[32]. The basis of the approach is to explore the *contexts* which may affect MBO decision-making (for example, location and nature of obstructing tumour, stages of disease, available treatments, care settings), the *mechanisms* or social processes through which decisions about treatment are negotiated (for example, collaboration or trust), and the *outcomes* of those decisions from both patient and clinical perspectives.

The review will address the following questions:

> ***Q1:*** *What is known about decision-making and its potential outcomes along current treatment pathways for malignant bowel obstruction?*
>
> ***Q2:*** *What constrains and enables decision-making in the treatment of MBO, according to the evidence, and how do these constraints and enablers compare with those described in shared decision-making (SDM) programme theory?*

The ultimate aim of a realist review is to build a tentative theoretical model (known as a 'programme theory') describing how a complex intervention or process might work in the practice environment, and test this against existing evidence. In the EPOC review, we will draw on existing models of shared decision-making to specify its components (see Figure 1), then compare these components with evidence of decision-making processes undertaken during the management of MBO. This process involves the gathering of 'fragments' of evidence[29], focusing not only on the research literature, but on any relevant documents connected with the phenomenon in question (such as clinical guidelines), taking a pragmatic and selective approach in line with resources and capacity for the review process.

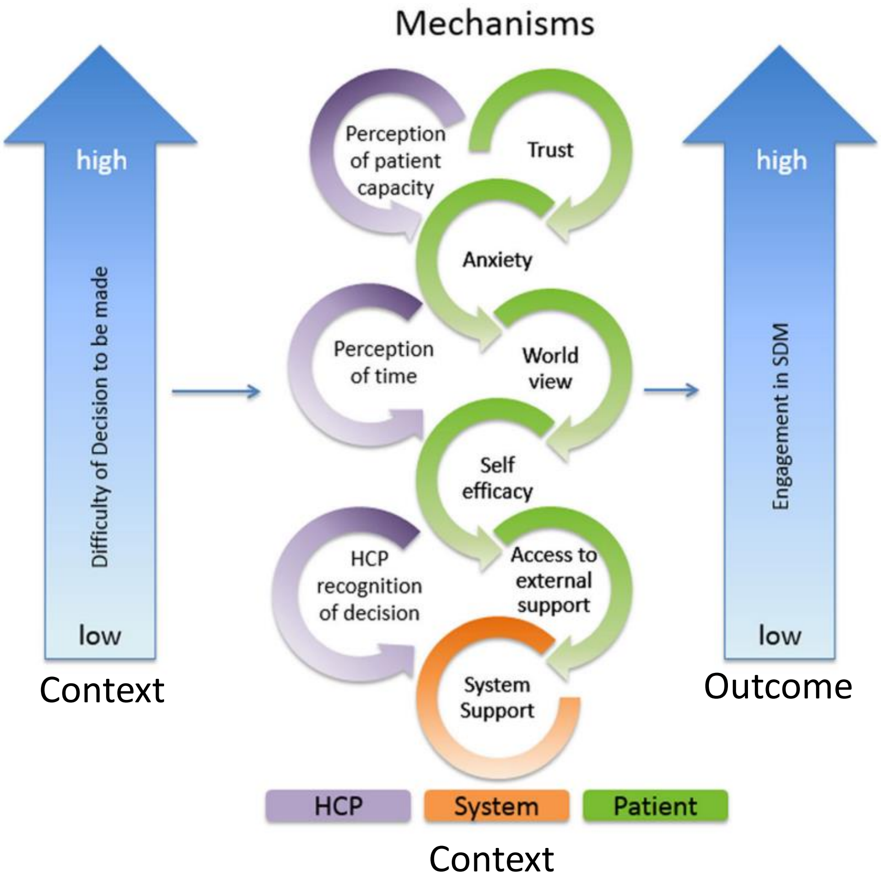

Figure 1 *A theory of shared decision-making from a literature review by Waldron et al[30], showing the processes or mechanisms which affect the way in which it is implemented.*

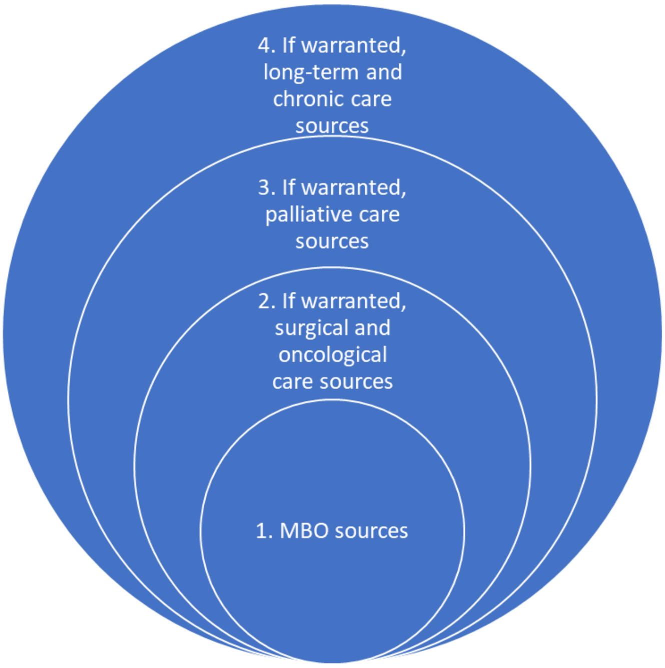

Figure 2 *An illustration of iterative searching to enable the refinement of a mid-range theory of shared decision-making into a programme theory of SDM in MBO.*

Searching is an iterative process, and searching strategies and associated methodologies for mapping findings will evolve in response to the findings at each stage. We anticipate that several searches might be required, to incorporate evidence from practice into the theory. The review will begin with a search for theories of shared decision-making, and will then synthesise the theoretical concepts in the SDM literature with the literature on shared and patient-centred decision-making in MBO to formulate a programme theory of SDM in MBO. We will then widen out to incorporate theoretical and practical evidence around shared decision-making in cancer care, palliative care and chronic illness to fill in any gaps in the theory suggested by the synthesis (see Figure 2).

We intend to combine robust and transparent systematic searching with the flexibility needed for a realist review. Search methods and resources might include but not be limited to:

- Electronic searches of major health databases (e.g. Medline, Embase and APA PsycInfo via OVID, CINAHL via EBSCOhost, CENTRAL AND CDSR via The Cochrane Library). Electronic searches of multidisciplinary databases (e.g. Web of Science, Google Scholar).
- Searches of specialist databases, grey literature and web resources (HMIC, Google searching, websites of relevant organizations active in this area to identify unpublished material, reports, policy documents, blogs etc).
- Supplementary searches including forward and backward citation searching of key and emerging citations, identifying "clusters" of data and citation networks from related publications.
- Contact with authors and stakeholders.

Database searches will be developed by an experienced information specialist examining the indexed terms (for example MeSH in Medline records) and free text words in previously identified relevant key papers and systematic reviews for the main concepts: malignant bowel obstruction (MBO) and decision-making and any associated concepts (e.g. broadening out into surgical and oncological care, and palliative care decision-making if warranted).

We will use search tools such as PubMed PubReminer to analyse search results and text to identify frequently used search terms, authors, journals relevant to the review's main concepts and tools like Pub Venn to visualise the relative size of search concepts and the overlap between them. The main search will be initially developed in OVID Medline. We will test the recall of major search concepts against known relevant reference sets (for example, a previous systematic review involving a search for MBO as a concept), and refine the search accordingly to achieve a balance of sensitivity and specificity.

The aim of the EPOC review will be to tailor the initial shared decision-making model to demonstrate how shared decision-making might, or might not, work in practice along MBO treatment pathways. This model will then be taken forward into the next phase of the study and evaluated in practice, in relation to the experiences of patients, informal caregivers and clinicians.

Stakeholder consultation: This consultation will explore patient, caregiver and clinician perspectives on decision-making in MBO. It will take the form of one 3-hour face-to-face workshop or two 1.5 hour on-line workshops (dependent on pandemic circumstances) for the discussion of emerging review findings with stakeholders, to direct key concepts for exploration in Work Package 3. Clinician stakeholders will include general surgeons, oncologists, specialist nurses, palliative care specialists and dietitians. A lay summary of the review findings will be sent to delegates prior to the workshop.

Session structure:

- Presentation of results of Work Package 1.
- Question and answer session.
- Consideration of the programme theory, clearly illustrated in a visual, to identifying any gaps (concepts felt to be important to shared decision-making in MBO that stakeholders feel are missing from the models currently in the literature).
- Summary and final questions/comments.
- Systematic gathering of stakeholder feedback.

Stakeholders (n~8–15 people with experience of MBO for each workshop, patients, caregivers and clinicians) will be recruited *via* charity partners (e.g. Bowel Cancer UK,

Ovacome), university and funder PPI groups, the research team's professional networks and social media (e.g. Twitter). In addition to enabling stakeholder involvement in setting out detailed objectives for the interview study (Phase 2), this procedure also aims to create stakeholder investment in the intervention design process (Phase 3) through relationship-building.

4.2 PHASE 2: Patient Trajectories Interview Study

An in-depth interview study with practitioners, patients and informal caregivers will explore how decision-making happens in practice in the management of MBO across a range of contexts (e.g. patient trajectories, practitioner roles, and care settings). Our objective is to explore how the shared decision-making pathways in our programme theory happen (or don't happen) in practice, and to use this understanding to refine the theory. We will explore the following questions:

> ***Q3:*** *How might patients', caregivers' and clinicians' experiences of MBO treatment decision-making differ across a range of contexts (e.g. stage of disease, setting, role perspective)?*
>
> ***Q4:*** *What constrains and enables the sharing of decision-making in the treatment of MBO, in practice?*

Settings: Two tertiary cancer centres (Hull University Teaching Hospitals NHS Trust; Leeds Teaching Hospitals NHS Trust); a single specialist palliative care unit (Dove House Hospice, Hull).

Sample size: We will conduct in-depth interviews with patients (n~25–35), caregivers (n~15–20) and health care practitioners (n~20–30) using participant-led graphic elicitation techniques (see below), guided by the findings of Phase 1. Recruitment will extend across 15 months. We anticipate recruiting between 60 and 85 interview participants over that time. Based on our experience of interviewing MBO patients and clinicians in previous research[4], and the breadth of patient trajectories and uncertainties around treatment, we do not anticipate reaching a point of data saturation (when no new themes or codes relevant to our theoretical model arise) with less than 60 interviews.

Identification, recruitment and consent: Written informed consent will be required from all participants. Eligibility: i) Patients with current or previous MBO, stable enough to participate; ii) informal caregivers of someone with MBO; iii) clinicians caring for patients with MBO. A realist approach demands purposive sampling to maximise variability[29]; the study will recruit patients from all stages of the trajectory, experiencing partial or complete obstruction. Interviews will explore how interviewees understand and experience shared decision-making along the MBO trajectory.

Theoretical framework: The interview study is grounded in a social constructionist epistemology[33], which is commensurate with realist philosophy. This takes the view that it is possible to gather, through participants' observations of the 'real' world, multiple perspectives on shared decision-making, which is a human activity facilitated through social processes. The epistemological standpoint of realist philosophy is that we can study this process through people's accounts of their experiences. Within this epistemology, we also draw on symbolic interactionism[34,35], a socio-psychological perspective that focuses on interpersonal relations and how these shape the behaviours of individual people through their ability to interpret and evaluate their social interactions.

Data collection: Qualitative interviews will use graphic elicitation techniques. Graphic elicitation is the use of diagramming in interviews to represent 'real life' experiences – interviewees create a simple diagram following a basic format explained by the researcher. This is a tried and tested method in health research, with roots in social work and psychological therapy[36,37]. It can reduce the burden of interviewing for patients who are very unwell[38] and the time-burden for clinicians, allowing the quick and focused representation of collaborative working around an episode of care and aiding recall[37,38,39]. Two techniques will be used: timelining and the Pictor technique.

Timelining: Timelines prompts interviewees to map significant events along a chronological line and explore each episode and the connections between them[36]. This can be combined with an axis to focus on eliciting fluctuating aspects of subjective experience over time. See Figure 3.

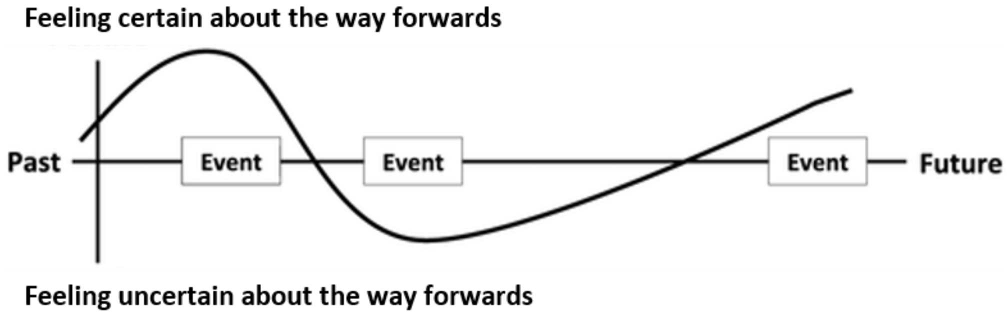

Figure 3 *Timelining: interview participants are asked to map significant events, such as symptoms or decision-making, along a line. Axes can facilitate dialogue about subjective feelings (e.g. certainty or uncertainty) using a continuous line mapped against events across the timeline.*

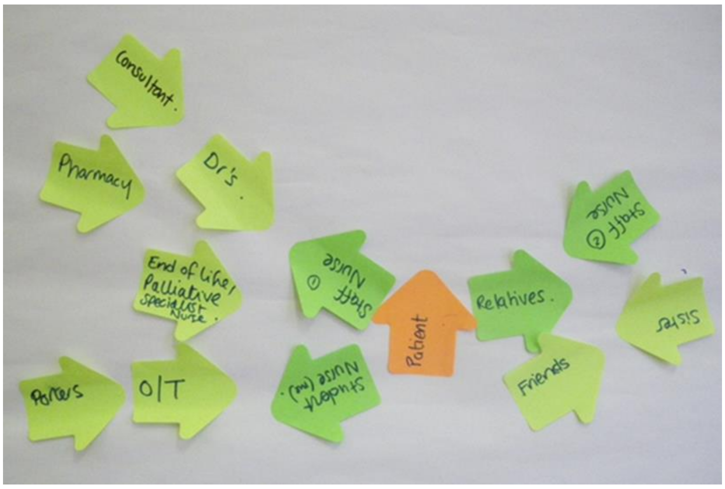

Figure 4 *A Pictor chart by a nurse, describing a 'tug of war' over goals of care for a cancer patient (left). Pictor in action (below). Pictor and timelining can be used in combination (below left).*

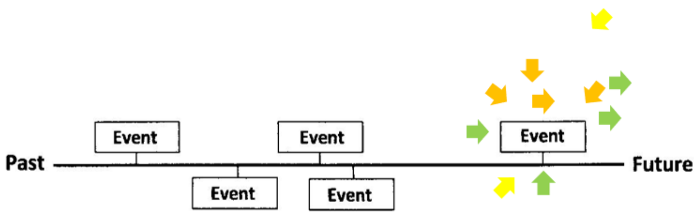

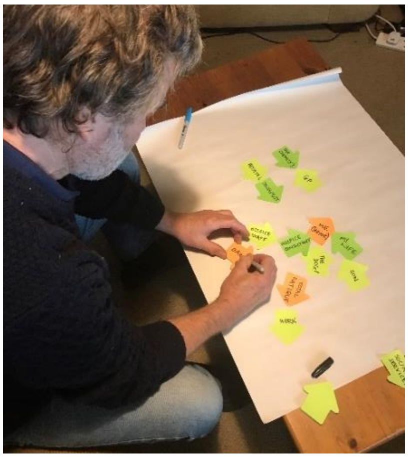

The Pictor technique: Pictor uses arrows to represent people and relationships. The interviewee is given a stack of semi-adhesive arrow-shaped Post-It™ notes and an A1 sheet of paper. They write down the role of each person or group involved in an episode of care on a separate arrow, and arrange the arrows on the paper to represent the 'story' of the event. The direction of the arrows and space between them are used to characterise interactions, and can be reconfigured during the interview. Pictor has proved particularly valuable in exploring health care collaboration in cancer and end-of-life care[38,39,40,41]. It can be used in combination with a timeline. See Figure 4.

Timelining will be used to explore the experiences of people whose experience is based on the management of recurrent or multiple bowel obstructions, with Pictor used to explore a single episode of decision-making depicted along the timeline where appropriate. Pictor alone will be used to explore the experiences of people who have experienced or managed a single episode of obstruction. It is anticipated that interview recruitment will continue over 15 months. If pandemic circumstances do not allow face-to-face interviews, or if interviewees are uncomfortable meeting face-to-face, interviews can be conducted by telephone (our previous research into MBO[4] demonstrated that patients with MBO are willing to conduct interviews by telephone, and that this can be done sensitively). Instruction for the preliminary construction of a timeline or Pictor chart can be sent through the post with the materials required, and it will be the interviewee's choice whether to construct a visual or conduct a conventional interview with no visual.

Analysis: Interviews will be audio-recorded and transcribed, and diagrams photographed. Analysis will combine grounded theory[42] (focusing on social processes) with configurational analysis[29] (focusing on links between context, social processes and outcomes), to enable us to refine the theory of shared decision-making.

4.3 PHASE 3: Intervention development

Findings of the review and interview study will be combined to present a model of shared decision-making in MBO in two face-to-face workshops with patients, informal caregivers and clinicians (this is scheduled for 2024; it will be conducted via on-line conferencing if pandemic conditions do not allow a face-to-face meeting). We will retain stakeholders from Phase 1 of the study where possible. The workshops will help us to refine our theory of shared decision-making in MBO by addressing the following question:

- *What are the core components of consistent patient-centred decision-making about treatment and goals of care across the trajectory of MBO?*

The workshops will follow the principles of experience-based co-design (ECBD), a process designed to enable patients and health care staff to work together to prioritise areas for improvement in health care services[31]. This process works with patient and staff experiences and the subjective feelings associated with these experiences in practice, rather than people's opinions about how things *should* work. In the EPOC study, the process will involve feeding back the findings of Phases 1 and 2 of the study and exploring how patient and caregiver experiences described in interviews informed or refined the theory produced by our initial review. We will use visual representations of pathways of decision-making based on the experiences of our interview study participants, and qualitative data describing those experiences, as a basis for the discussion of:

- how decision-making happens in practice;
- what its core components are in relation to MBO and its trajectory;
- when/how decisions might be made in partnership between clinician and patient;
- how best to ensure that the goals of care remain patient-centred across all potential treatment pathways.

The aim of the stakeholder consultations and the co-design process will be to produce theoretically-driven content for interventions to assist in the management of MBO patients in two formats: print and/or digital information and decision aids for patients and caregivers and digital learning tools for clinicians to be hosted by the NHS eLearning platform.

5 Project Management

The study will be guided by a Project Management Group (meeting monthly) and an independent Project Steering Group (meeting bi-annually), and will be subject to audit/monitoring according to Sponsor and site R&D requirements and funder requirements (via six-monthly Key Performance Indicator reports to Yorkshire Cancer Research).

6 Patient and Public Involvement

Two project-specific patient and caregiver partners with experience of MBO as a patient/caregiver have been engaged, one as part of the bi-annual Steering Management Group (SMG, Role 1) and another as part of the monthly Project Management Group (PMG, Role 2). An outline of the tasks to be undertaken were agreed with both, and the agreed tasks are shown in Figure 5. Members of the *Hull INVOLVE* Patient and Public Involvement group (a University of Hull PPI network) will also be engaged to consult on the wording of public-facing study documentation. PPI representatives will be paid for their time.

Charity partnerships: Two charities have agreed to act as partners for the project, Bowel Cancer UK and Ovacome ovarian cancer charity, to publicise the study and engage stakeholders for consultation as appropriate to the work phases described above.

7 Ethical and Regulatory Approvals

The EPOC study will be conducted according to the principles of good research practice outlined by the Research Governance Framework for Health and Social Care. Sponsorship for the study will be arranged via the University of Hull. Preliminary approvals will be sought from the Hull York Medical School Ethics Panel, and subsequently from the NHS Research Ethics Committee (REC) and the Health Research Authority (HRA) prior to recruitment to the interview study via acute and palliative care settings. The identity of interview participants will be preserved in accordance with the Data Protection Act (2018) and GDPR (2018).

ROLE 1: PPI Contributor to EPOC Steering Management Group

Aim: To contribute to the development of the study by ensuring that the perspective of patients and caregivers and the quality of patient care are central considerations throughout, and that patient-relevance is a central concern in the evidence-based interventions produced by the study.

Activities:

- Prepare for steering meetings by reading and considering all papers circulated before the meeting.
- Attend 6-monthly steering meetings to provide a patient/caregiver/ public perspective to guide the direction of the study.
- Make suggestions that will contribute to the successful recruitment of patients and caregivers to stakeholder workshops and qualitative interviews.
- Make suggestions that will improve the experience of those being recruited to the study.
- Make suggestions which will improve the depth and quality of information coming from the interviews and stakeholder groups.
- Contribute to discussions on the analysis of results.
- Suggest channels through which the results of the study can be effectively communicated to concerned patient and caregiver groups and interested members of the public.
- Take part in the dissemination of the results of the study to enhance its reach and impact.
- Monitor the compliance of the PPI elements of the study with the UK Standards for Public Involvement.

ROLE 2: PPI Contributor to EPOC Project Management Group

Aim: To contribute to the development of the study by ensuring that the perspectives of patients and caregivers and the quality of patient care are central considerations throughout, and that study documentation and dissemination reflects this.

Activities:

- Co-design public-facing documentation by commenting on the language and layout of draft documents prepared by the research team.
- Work with the lead researcher to create the best strategy for contributing to and attending monthly project management meetings.
- Attending *ad hoc* PPI meetings on-line, convened at your convenience, where this is agreed to be the best strategy for maximising your contribution.
- Prepare for meetings by reading and considering all papers circulated before the meeting.
- Discuss and refine the strategy for recruitment of patient/caregiver interviewees and stakeholder workshop members with the lead researcher, and make suggestions to encourage attention to cultural diversity in recruitment processes for interviews and stakeholder workshops.
- Help develop questions to be asked of interviewees in each group.
- Contribute to development of lay study results for public consumption
- Suggest channels through which the results of the study can be effectively communicated to concerned patient and caregiver groups and interested members of the public.
- Contribute to development of lay information resources for patients /caregivers.
- Take part in the dissemination of the results of the study to enhance its reach and impact.

Figure 5 *Agreed public engagement roles.*

8 Data Management and Archiving

Anonymised audio, text and visual methods data will be uploaded to a secure private channel Microsoft Teams at the university of Hull and will be available only to members of the research team. Personal contact data for stakeholders and interviewees will be kept in a password protected Excel file accessible only to the Principal Investigator and Study Administrator. Written consent forms will be kept in a locked cabinet at Hull York Medical School (Allam Building, University of Hull).

9 Study Timeline

2021			
**SEPT**	**OCT**	**NOV**	**DEC**
Study set-up	**Realist review of the literature & NHS documents**		
	Plan strategy	Commence searches	Search/Analysis
		**Ethics applications in**	

2022											
**JAN**	**FEB**	**MAR**	**APR**	**MAY**	**JUN**	**JUL**	**AUG**	**SEPT**	**OCT**	**NOV**	**DEC**
**Realist review of the literature & NHS documents**											
Search/analysis	Prepare theory of decision-making	**Stakeholder consultation**	Search/analysis		Refine theory of decision-making	**Stakeholder consultation**	**Review write-up**				
**Ethics approvals process**											
				Patient trajectories interview study: Recruitment begins via charity partners/NHS						**Interview study**	

2023											
**JAN**	**FEB**	**MAR**	**APR**	**MAY**	**JUN**	**JUL**	**AUG**	**SEPT**	**OCT**	**NOV**	**DEC**
**Interview study** Including transcription and qualitative analysis											

2024							
**JAN**	**FEB**	**MAR**	**APR**	**MAY**	**JUN**	**JUL**	**AUG**
**Intervention development**							
Preparation	Stakeholder workshops	Development and production of interventions					
						Dissemination	

10 References

1.Winner M, Mooney SJ, Hershman DL, Feingold DL, Allendorf JD, Wright JD, Neugut AI. Incidence and predictors of bowel obstruction in elderly patients with stage IV colon cancer: A population-based cohort study. JAMA surgery. 2013; 148(8):715-722.

2. Tuca A, Guell E, Martinez-Losada E, Codorniu N. Malignant bowel obstruction in advanced cancer patients: epidemiology, management, and factors influencing spontaneous resolution. Cancer Management Research. 2012; 4:159e169.

3. Mercadante S. Intestinal dysfunction and obstruction. In: Walsh D (Ed.) Palliative medicine. Philadelphia, PA: Saunders/Elsevier. 2009; 1267e1275.

4. Baddeley E & Bravington A, Johnson M, Currow DC, Murtagh FEM, Boland E, Obita G, Nelson A, Seddon K, Oliver A, Noble S, Boland J. Development of a core outcome set to use in the research and assessment of malignant bowel obstruction: protocol for the RAMBO study. BMJ Open. 2020; 10:e039154.5.

5. Gwilliam B, Bailey C. The nature of terminal malignant bowel obstruction and its impact on patients with advanced cancer. International Journal of Palliative Nursing. 2001; 7(10):474-481.

6. Platt V. Malignant bowel obstruction: so much more than symptom control. International Journal of Palliative Nursing. 2001; 7(11):547-554.

7. Cousins SE, Tempest E, Feuer DJ Cousins SE, Tempest E, Feuer DJ. Surgery for the resolution of symptoms in malignant bowel obstruction in advanced gynaecological and gastrointestinal cancer. Cochrane Database of Systematic Reviews. 2016; Issue 1. Art. No.: CD002764. doi: 10.1002/14651858.CD002764.pub2

8. Feuer DJ, Broadley KE. Corticosteroids for the resolution of malignant bowel obstruction in advanced gynaecological and gastrointestinal cancer. Cochrane Database of Systematic Reviews. 2000; Issue 1. Art. No.: CD001219. doi: 10.1002/14651858.CD001219

9. Sowerbutts AM, Lal S, Sremanakova J, Clamp A, Todd C, Jayson GC, Teubner A, Raftery AM, Sutton EJ, Hardy L, Burden S. Home parenteral nutrition for people with inoperable

malignant bowel obstruction. Cochrane Database of Systematic Reviews. 2018; Issue 8. Art. No.:CD012812. doi: 10.1002/14651858.CD012812.pub2

10. Obita GP, Boland EG, Currow DC, Johnson MJ, Boland JW. Somatostatin analogues compared with placebo and other pharmacologic agents in the management of symptoms of inoperable malignant bowel obstruction: A systematic review. Journal of Pain and Symptom Management. 2016; 52(6):901-19.e1. doi: 10.1016/j.jpainsymman.2016.05.03211.

11. Paul Olson TJ, Pinkerton C, Brasel KJ, Schwarze ML. Palliative surgery for malignant bowel obstruction from carcinomatosis. JAMA Surgery. 2014; 149(4): 383-392.

12. Merchant SJ, Brogly SB, Booth CM, Goldie C, Peng Y, Nanji S, Patel SV, Lajkosz K, Baxter NN. Management of cancer-associated intestinal obstruction in the final year of life. Journal of Palliative Care. 2020; 35(2):84-92.

13. Moga M, Blidaru A, Casap S, Pascu A, Cobelschi C, Dima L. Medical management versus palliative surgery for bowel obstruction in ovarian cancer. Gineco.eu. 2014; 10:117-119.

14. Burch J, Tort S (on behalf of Cochrane Clinical Answers Editors). What are the benefits and harms of surgery for malignant bowel obstruction? Cochrane Clinical Answers. 2018; doi: 10.1002/cca.1946

15. Singh Curry R, Evans E, Raftery AM, Hiscock J, Poolman M. Percutaneous venting gastrostomy/gastrojejunostomy for malignant bowel obstruction: a qualitative study. BMJ supportive & palliative care. 2019; 9(4):381-8.

16. Sowerbutts AM, Lal S, Sremanakova J, Clamp AR, Jayson GC, Teubner A, et al. Palliative home parenteral nutrition in patients with ovarian cancer and malignant bowel obstruction: experiences of women and family caregivers. BMC Palliat Care. 2019;18(1):120.

17. Ferguson HJM, Ferguson CI, Speakman J, Ismail T. Management of intestinal obstruction in advanced malignancy. Annals of Medicine and Surgery. 2015; 4:264-270.

18. Parker SM, Clayton JM, Hancock K, Walder S, Butow P, Carrick S, Currow D, Ghersi D, Glare P, Hagerty R, Tattersall MHN. A systematic review of prognostic/end-of-life communication with adults in the advanced stages of a life-limiting illness: Patient/caregiver

preferences for the content, style, and timing of information. Journal of Pain and Symptom Management. 2007; 34(1):81-93.

19. Michael A, Allan L, Bennett-Eastley K, Herbertson R, Skene SS. Edmond: a feasibility study of elemental diet as an alternative to parenteral nutrition for ovarian cancer patients with inoperable malignant bowel obstruction. International Journal of Gynaecological Cancer. 2020; 30(4): A85.1-A85. doi: 10.1136/ijgc-2020-ESGO.144

20. Bethune R, Sbaih M, Brosnan C, Arulampalam T. What happens when we do not operate? Survival following conservative bowel cancer management. Annals of the Royal College of Surgeons England. 2016; 98:409-412.

21. Oh SY, Jun HJJ, Park SJ, Park K, Lim GJ, Yu Y, Cho s-J, Song A. Randomized phase II study to assess the effectiveness of fluid therapy or intensive nutritional support on survival in patients with advanced cancer who cannot be nourished via enteral route. Journal of Palliative Medicine. 2014; 17(11): 1266-1270.

22. Shariff F, Bogach J, Guidolin K, Nadler A. Malignant bowel obstruction management over time: Are we doing anything new? A current narrative review. Ann Surg Oncol. 2021; on-line at https://doi.org/10.1245/s10434-021-10922-1

23. NICE. Improving supportive and palliative care for adults with cancer: Cancer service guideline CSG4. London/Manchester: National Institute for Health and Care Excellence. 2004.

24. Houska A, Tuckova A, Vlckova K, Polakova K, Loucka M. Optimal participation in decision-making in advanced chronic disease: perspectives of patients, relatives and physicians. Annals of Palliative Medicine. 2021; apm-20-2368

25. Kousmanen L, Hupli M, Ahtiluoto S, Haavisto E. Patient participation in shared decision-making in palliative care - an integrative review. Journal of Clinical Nursing. 2021; 00:1-14.

26. Belanger E. Shared decision-making in palliative care: Research priorities to align care with patients' values. Palliative Medicine. 2017. 31(7):585-586.

27. Ellis-Smith C, Tunnard I, Dawkins M, Gao W, Higginson IJ, Evans C. Managing clinical uncertainty in older people towards the end of life: a systematic review of person-centred tools. BMC Palliative Care. 2021; 20:168-209.

28. Pawson R. *The science of evaluation: A realist manifesto.* 2013; London: Sage.

29. Emmel N, Greenhalgh J, Manzano A, Monaghan M, Dalkin S. (Eds) *Doing realist research.* 2018; London: Sage.

30. Waldron T, Carr T, McMullen L, Westhorp G, Duncan V, Neufeld S-M, Bandura L-A, Groot G. Development of a program theory for shared decision-making: a realist synthesis. BMC Health Services Research. 2020; 20:59.

31. The Point of Care Foundation. Experience-based co-design toolkit. 2016. Accessed on 13.1.22 at https://www.pointofcarefoundation.org.uk/resource/experience-based-co-design-ebcd-toolkit/

32. Greenhalgh T, Wong G, Westthorp G, Pawson R. Protocol – realist and meta-narrative evidence synthesis: Evolving Standards (RAMESES). *BMC Medical Research Methodology.* 2011; 11:115-125.

33. Burr V. *Social constructionism.* 2nd ed. London: Routledge; 2003.

34. Mead GH. *Mind, self and society.* Chicago: University of Chicago Press; 1934/2015.

35. Blumer H. *Symbolic interactionism: Perspective and Method.* California: University of California Press; 1967.

36. Bravington A and King, N. Putting graphic elicitation into practice: Tools and typologies for the use of participant-led diagrams in qualitative research interviews. *Qualitative Research.* 2019; 19(5):506-523.

37. Hargreaves, C. P. (1979). Social networks and interpersonal constructs. In P. Stringer & D. Bannister (Eds.), Constructs of sociality and individuality (pp. 153-175). London: Academic Press.

38. Hardy B, King N, Firth J. Applying the Pictor technique to research interviews with people affected by advanced disease. *Nurse Researcher.* 2012; 20(1):6-10.

39. King N, Bravington A, Brooks J, Hardy B, Melvin J, Wilde D. The Pictor technique: A method for exploring the experience of collaborative working. *Qualitative Health Research.* 2013; 23(8): 1138-1152.

40. Johnston, B., Matthews, G., Patterson, A., Bravington, A., Hardy, B., Seymour, J. Qualitative component of a longitudinal mixed methods programme evaluation using in-depth interviews. *BMJ Supportive and Palliative Care. 2018;* 8:suppl 1_ A1-79.

41. Ross A, King N, Firth J. Interprofessional relationships and collaborative working: Encouraging reflective practice. *Online Journal of Issues in Nursing*. 2005; 10(1):Art.3.

42. Charmaz K. *Constructing grounded theory.* 2nd Ed. 2014; London: Sage.
